# Synthesis of a 2D Cu@TiO_2_ composite *via* the design of a 1D Cu-based coordination polymer precursor for efficient and selective photodegradation of dyes†

**DOI:** 10.1039/d1ra09309f

**Published:** 2022-03-24

**Authors:** Ya-Qian Zhang, Ning Xu, Yu Liu, Xiao-Sa Zhang, Wen-Ze Li, Hong-Tian Zhao, Jian Luan

**Affiliations:** College of Science, Shenyang University of Chemical Technology Shenyang 110142 P. R. China liwenze@syuct.edu.cn; College of Sciences, Northeastern University Shenyang 100819 P. R. China 2010044@stu.neu.edu.cn; College of Chemistry, Liaoning University Shenyang 110036 P. R. China

## Abstract

A 2D Cu@TiO_2_ composite with a porous and crystalline structure was successfully synthesized *via* one-step and low-temperature calcination of a 1D Cu-based coordination polymer (Cu-CP), namely [Cu_2_(3-dpha)(1,4-NDC)_2_(H_2_O)_3_]_*n*_ (3-dpha = *N*,*N*′-bis(3-pyridyl)adipamide and 1,4-H_2_NDC = 1,4-naphthalenedicarboxylic acid). Moreover, the Cu@TiO_2_ membrane was fabricated by a simple filtration of the as-grown Cu@TiO_2_ composite. Compared with the benchmark TiO_2_ photocatalyst, the Cu@TiO_2_ composite material with high specific surface area and reduced photogenerated electron–hole ratio exhibited good photodegradation activity and durability for gentian violet (GV), which could be attributed to the combined effect of co-doping of Cu and TiO_2_ structure. Furthermore, the ˙OH and ˙O_2_^−^ radicals were predicted to dominate the photocatalytic process. Therefore, this new efficient photocatalyst is a promising candidate for efficient and selective photodegradation of organic pollutants.

## Abstract 2

A 2D Cu@TiO_2_ composite was synthesized via low-temperature calcination of a 1D Cu-CP. Moreover, the Cu@TiO_2_ membrane was fabricated by a simple filtration of the Cu@TiO_2_ composite which exhibited selective photodegradation activity for GV.

## Graphical abstract

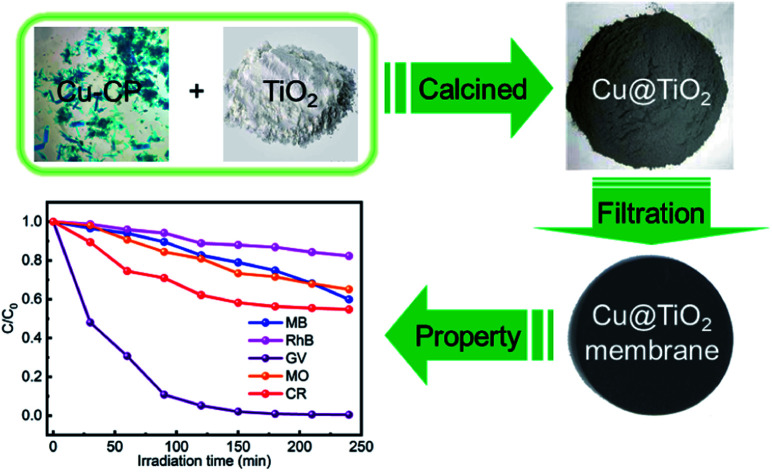

## 1. Introduction

Industrial wastewater with various organic pollutants causes hazard to human health and the ecological environment.^1^ To remove the organic pollutants in industrial wastewater, various methods have been explored, including physical adsorption,^2^ chemical adsorption,^3^ biological degradation,^4^ and photocatalysis.^5^ Unlike traditional treatment methods, photocatalysis can remove low concentration contaminants without secondary treatment.^6–9^ Among numerous photocatalysts, TiO_2_ has been considered as the most promising photocatalyst, attributable to its chemical stability, non-toxicity, affordability, environmental friendliness and high catalytic activity.^10–13^ Unfortunately, the photocatalytic efficiency of pure TiO_2_ is quite small because of its large bandgap, it is more prone to excitation upon UV irradiation and the rate of recombination of photogenerated electron/hole (e^−^/h^+^) pairs is high.^14^ Therefore, a model photocatalyst should exhibit a bandgap suitable for excitation by UV light. The ideal photocatalysts should possess good charge separation efficiencies and suitable positions of the valence band (VB) and conduction band (CB) for redox reactions with nanostructures.^15^

In this regard, several structural and chemical modifications have been adopted to enhance the photocatalytic activity of TiO_2_.^16–19^ Particularly, doping with metals is considered to be an effective method for increasing the photocatalytic efficiency of TiO_2_ as this impedes e^−^/h^+^ recombination and enhances its UV-light harvesting capacity.^18,19^ Metallic elements can induce a suitable bandgap shift and promote recombination, as a result of minimizing the photocatalysis capability in combination with thermal instability.^20,21^ Among them, noble metal doping for TiO_2_ is generally considered to be a good choice for enhancing the photocatalytic activity of TiO_2_.^20,21^ However, the reaction setup is costly and the method is hampered by several other drawbacks as noble metals like Au and Ag are quite toxic in nature.^22^ Another drawback of using noble metal-doped photocatalysts is that they can undergo photo-corrosion during the photocatalytic process.^22^ Hence, using transition metals, such as Co, Ni and Cu, instead of noble metals to dope TiO_2_ photocatalysts seems to be a viable approach for developing photocatalysts.^23,24^

**Scheme 1:**
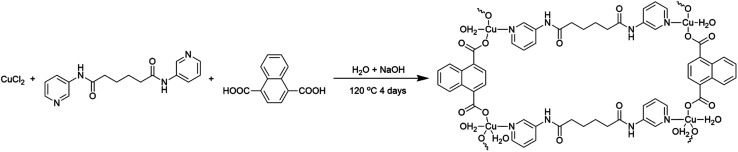
Schematic illustration for the synthesis of the Cu-CP.

**Scheme 2:**
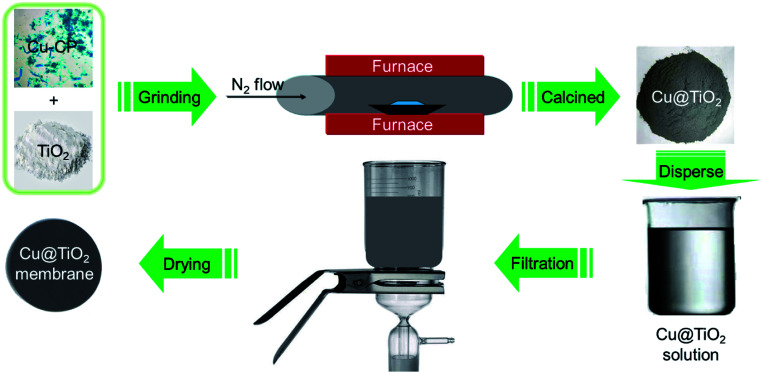
Schematic illustration for the synthesis of 2D Cu@TiO_2_ membrane derived from 1D Cu-CP.

Recently, coordination polymers (CP), comprising of metal centers and coordinated organic linkers, are an emerging class of porous materials that attract intensive attentions for their highly adjustable pore size and shape, diverse structures and various functionalities.^25–28^ Synthesis of transition metal doped TiO_2_ from CPs is considered as a promising process.^29^ Moreover, CPs are utilized as sacrificial precursors for forming the uniform distribution of elements, large specific surface area, highly dispersed active sites and controllable compositions.^25–28^ Herein, we synthesized the Cu@TiO_2_ hybrid *via* simple calcination of Cu-CP (Scheme 1) at low temperature and investigated their good photodegradation efficiency *via* gentian violet (GV) induced by UV light. Moreover, it is worth noting that most of the developed adsorbents are powders, and this leads to serious recontamination and recycling problems. From this respect, membrane materials are highly desired, but this usually comes with the problems of reduction in both photodegradation ability and stability in solutions. Herein, a high purity Cu@TiO_2_ membrane was fabricated by simple filtration of Cu@TiO_2_ hybrid (Scheme 2). The Cu@TiO_2_ membrane showed good stability in solutions. We explored this membrane as a photocatalyst for the photodegradation of GV without involving any other complex process. The membrane showed excellent selective and repeatable photodegradation for GV with a record high photodegradation of 99.51% and a stability that showed no structure and performance degradation after 10 cycles under UV light irradiation. To the best of our knowledge, in the case of CP-derived TiO_2_ doping, there is no example in which the metal-doped TiO_2_ materials did enhance the photodegradation performances of their parent materials under UV light.

## 2. Experimental section

### 2.1 Materials

All reagents and solvents employed for this synthesis were purchased from commercial sources and used as received without further purification. The elemental analyses (C, H, and N) were determined with a PerkinElmer 240C elemental analyzer. Infrared spectra were recorded with a Varian 640 FT-IR spectrometer over the range of 500–4000 cm^−1^ with the use of KBr pellets as sample matrices. Powder X-ray diffraction (PXRD) data was collected using a Rigaku diffractometer with Cu Kα radiation. Thermogravimetric analysis (TGA) measurements were performed with a METTLER TOLEDO thermal analyzer at a heating rate of 5 °C min^−1^ under a N_2_ atmosphere. The morphology and structure of the sample was characterized *via* scanning electron microscopy (SEM, Nova Nano SEM 430) and high resolution transmission electron microscopy (HRTEM, JEOL 2010 at 200 kV). The specific surface area and pore structure of the sample was investigated with an automatic volumetric sorption analyzer (ASAP 2020 M) using N_2_ as the adsorbate at −196 °C. X-ray photoelectron spectroscopy (XPS) was performed using an Escalab 250 with an Al Kα radiation. UV-Vis absorption spectra were recorded with the use of an SP-1900 UV-Vis spectrophotometer.

### 2.2 Synthesis of [Cu_2_(3-dpha)(1,4-NDC)_2_(H_2_O)_3_]_*n*_ (1)

The mixture of CuCl_2_·2H_2_O (0.034 g, 0.20 mmol), *N*,*N*′-bis(3-pyridyl)adipamide (3-dpha, 0.031 g, 0.10 mmol), 1,4-naphthalenedicarboxylic acid (1,4-H_2_NDC, 0.032 g, 0.15 mmol), H_2_O (12 mL), and NaOH (0.016 g, 0.40 mmol) was stirred for 30 min in air, and then transferred and sealed in a 25 mL Teflon reactor, which was heated at 120 °C for 4 days. After slow cooling to room temperature, blue block crystals of 1 were obtained in 30% yield based on Cu. C_40_H_36_Cu_2_N_4_O_13_ (907.81): calcd C 52.92, H 4.00, N 7.05; found C 52.96, H 3.99, N 7.03. IR (KBr, cm^−1^): 3454 (s), 3071 (w), 2925 (w), 2854 (w), 1704 (w), 1615 (w), 1549 (s), 1479 (w), 1373 (m), 1272 (w), 1298 (w), 1148 (w), 915 (w), 774 (w), 693 (w), 575 (w).

### 2.3 Synthesis of Cu@TiO_2_ membrane

First of all, a mixture containing complex 1 (100 mg) and TiO_2_ powder (50 mg) were evenly grinded in solid phase, and was placed in a quartz boat and inserted into a tubular furnace. Subsequently, the furnace temperature was raised to 360 °C over the span of 40 min under a nitrogen flow (200 mL min^−1^, purity 99.999%). The metal-based composite (that is, Cu@TiO_2_) was obtained by continuous heating under a nitrogen flow (200 mL min^−1^) for 60 min. After the synthesis of Cu@TiO_2_ was complete, the reactor was cooled to room temperature under a nitrogen flow. The Cu@TiO_2_ membrane was fabricated by a simple filtration method. About 20 mg of Cu@TiO_2_ powders were ultrasonicated in 50 mL of an ethanol solution. The suspension was then filtered using a porous cellulose membrane filter with a pore diameter of 0.42 mm. The thickness of the membrane is about 20 μm with poor mechanical properties. When the membrane is removed from the filter, the membrane collapses itself.

### 2.4 Dye photocatalytic experiments

The photocatalytic activities of the prepared complex 1 and Cu@TiO_2_ composite (5 mg) were evaluated by decomposing five dyes (methylene blue (MB), rhodamine B (RhB), methyl orange (MO), Congo red (CR), and gentian violet (GV)) in aqueous solutions (100 mL, 10 mg L^−1^ for MB, RhB and GV, 40 mg L^−1^ for MO, and 80 mg L^−1^ for CR) under illumination with UV light. During the photocatalytic experiment, an identical suspension to that described above was continuously stirred for at least 60 min in the dark in order to reach adsorption–desorption equilibrium prior to the of UV light source. After attaining adsorption–desorption equilibrium between the five dyes and complex 1 or Cu@TiO_2_ composite, the suspension of the catalyst and five dyes were irradiated with a beam of light generated by a 20 W high pressure Hg lamp. The change in the absorbance during the observed time interval of the reaction in the aliquots was monitored with a UV-Vis spectrophotometer. The degradation value was computed *via*eqn (1):1
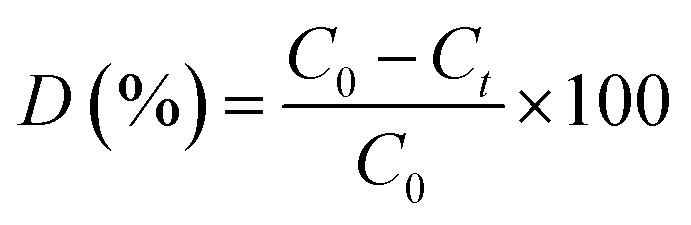
where *C*_0_ and *C*_*t*_ are the concentrations of the five dyes at *t* = 0 and after *t* minutes of photocatalytic reaction, respectively, while *D* denotes the degradation efficiency.

### 2.5 X-ray crystallography

X-ray diffraction data for complex 1 was collected with a Bruker SMART APEX II diffractometer with Mo Kα (*λ* = 0.71073 Å) irradiation source. This system was operated in the ω and θ scan modes. All of the structures were solved by direct methods and refined on *F*^2^ by full-matrix least-squares methods using the SHELXS program provided with the SHELXTL package.^30^ For complex 1, the crystal parameters, obtained data and refinement results are summarized in Table 1. Selected bond distances and bond angles are listed in Table S1 in the ESI.† Hydrogen bonding geometries of complex 1 is listed in Table S2 (ESI†). CCDC 2128376 file for complex 1 contains the supplementary crystallographic data supporting the results described in this paper.†

**Table 1:** Crystal and refinement data for complex 1

Empirical formula	C_40_H_36_Cu_2_N_4_O_13_
*F*_w_	907.81
Crystal system	Monoclinic
Space group	*P*2_1_/*n*
*a* (Å)	17.347(2)
*b* (Å)	10.9004(12)
*c* (Å)	19.960(2)
*α* (°)	90
*β* (°)	99.553(4)
*γ* (°)	90
*V* (Å^3^), *Z*, *T* (K)	3721.9(7), 4, 296(2)
*D*_c_/g cm^−3^, *F*(000)	1.620, 1864
Goodness-of-fit on *F*^2^	1.020
Reflections collected	58 529
Unique data, *R*_int_	9255, 0.0417
*θ* range (°)	2.07–28.33
*R*_1_ (*I* > 2*σ*(*I*))^a^	0.0614
w*R*_2_^b^ (all data)^a^	0.1158

a *R*_1_ = ∑‖*F*_o_| − |*F*_c_‖/∑|*F*_o_|.

b w*R*_2_ = ∑[w(*F*_o_^2^ − *F*_c_^2^)^2^]/∑[w(*F*_o_^2^)^2^]^1/2^.

## 3. Results and discussion

### 3.1 Structural description and characterization of [Cu_2_(3-dpha)(1,4-NDC)_2_(H_2_O)_3_]_*n*_ (1)

**Fig. 1:**
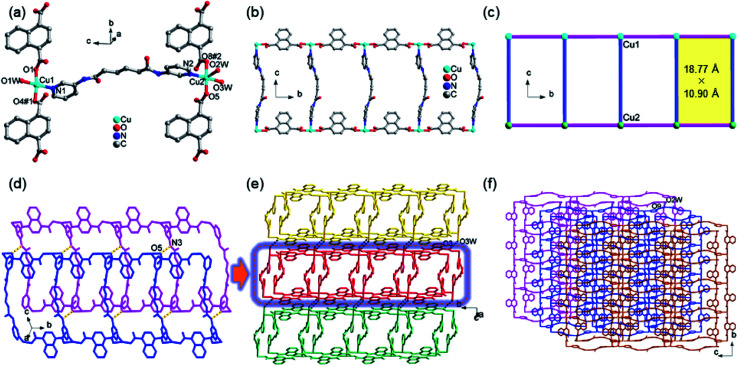
(a) The coordination environment of Cu(ii) center in complex 1; (b) the 1D ladder-like chain in 1. (c) A simplified view of the 1D chain in 1. (d) The dipolymer with N–H⋯O hydrogen bond formed between the amino nitrogen atom (N3) of 3-dpha and the carboxylic oxygen atom (O5) from 1,4-NDC anion in 1; (e) a view of the 2D supramolecular layer with O–H⋯O hydrogen bond in 1. (f) A view of the 3D supramolecular network with O–H⋯O hydrogen bond in 1.

The structure of 1 contains two crystallographically unique Cu(ii) ions, as depicted in Fig. 1a. The Cu1 ion is tetrahedral with the distortion parameter *τ*_4_ (0.31), calculated by the four-coordinate geometry index *τ*_4_ = {360° − (*α* + *β*)}/141,^31^ where *α* and *β* are the two largest angles of the tetrahedron, *via* coordinating to one pyridyl nitrogen atoms (N1) of 3-dpha, two oxygen atoms (O1 and O4#1) from two 1,4-NDC anions, and one oxygen atom (O1W) of a coordination water molecule. The square-pyramidal Cu2 center *τ*_5_ (0.13) defines the geometric parameter *τ*_5_ = (*β* − *α*)/60 (ref. 32) which is applicable to the five-coordinate structure as an index of the degree of trigonality, within the structural continuum between trigonal bipyramidal and rectangular pyramidal as well as Cu2 center is coordinated by one pyridyl nitrogen atoms (N2) of 3-dpha and two oxygen atoms (O5 and O8#2) of two 1,4-NDC anions plus two oxygen atoms (O2W and O3W) of coordination water molecules [Cu–N = 1.988(2) and 2.029(2) Å, Cu–O = 1.9265(17)–2.2214(18) Å]. The adjacent Cu1 or Cu2 centers are linked by the μ_2_-bridging 1,4-NDC anions to form a 1D [Cu(1,4-NDC)]_*n*_ linear chain (Fig. 1b), the non-bonding Cu⋯Cu distance is 10.90 Å. Then, each 3-dpha ligand acting as a bidentate ligand coordinates to the two linear chains with the non-bonding Cu⋯Cu distance is 18.77 Å, resulting in a ladder-like chain (Fig. 1c). Meanwhile, adjacent ladder-like chains were linked by N3–H3B⋯O5 (3.0036 Å, 162°) to form a dipolymer (Fig. 1d). Moreover, the hydrogen-bonding interactions between the coordinated water molecules and the carboxyl oxygen atoms O3W–H3WB⋯O3 (2.7628 Å, 162°) are found to assemble these dipolymers into a 2D layer (Fig. 1e). Additionally, the O2W–H2WA⋯O9 (2.7205 Å, 125°) hydrogen-bonding interactions interlink the adjacent layers to form a 3D supramolecular framework (Fig. 1f). The solvent-accessible voids by PLATON^33^ calculations are only 0.9% for 1.

The IR spectrum of complex 1 is shown in Fig. S1.† The bands at 3450 cm^−1^ is assigned to the stretching and bending vibrations of the –OH groups of water molecules for 1.^34^ The strong peaks at 1610 cm^−1^ for 1 is identified as the *ν*_C=O_ vibration of the amide group.^34^ The presence of the characteristic bands at 1550 and 1490 cm^−1^ for 1 may be attributed to the asymmetric and symmetric vibrations of the carboxyl groups.^34^ The strong peaks at 1380 and 1050 cm^−1^ for 1 suggest the *ν*_C–N_ stretching vibrations of the pyridyl ring of the 3-dpha ligands.^35^ The powder X-ray diffraction (PXRD) patterns for complex 1 are presented in Fig. S2.† The as-synthesized pattern is in good agreement with the corresponding simulated one, indicating the phase purity of the sample. Thermogravimetric analysis is carried out for complex 1 in order to investigate their thermal stability. As shown in Fig. S3,† the coordination water molecules and organic components of complex 1 decompose from 185 °C. The remaining residue corresponds to the formation of CuCO_3_ at 650 °C (obsd 27.37%, calcd 27.22%).

### 3.2 Structural description and characterization of the Cu@TiO_2_ material

**Fig. 2:**
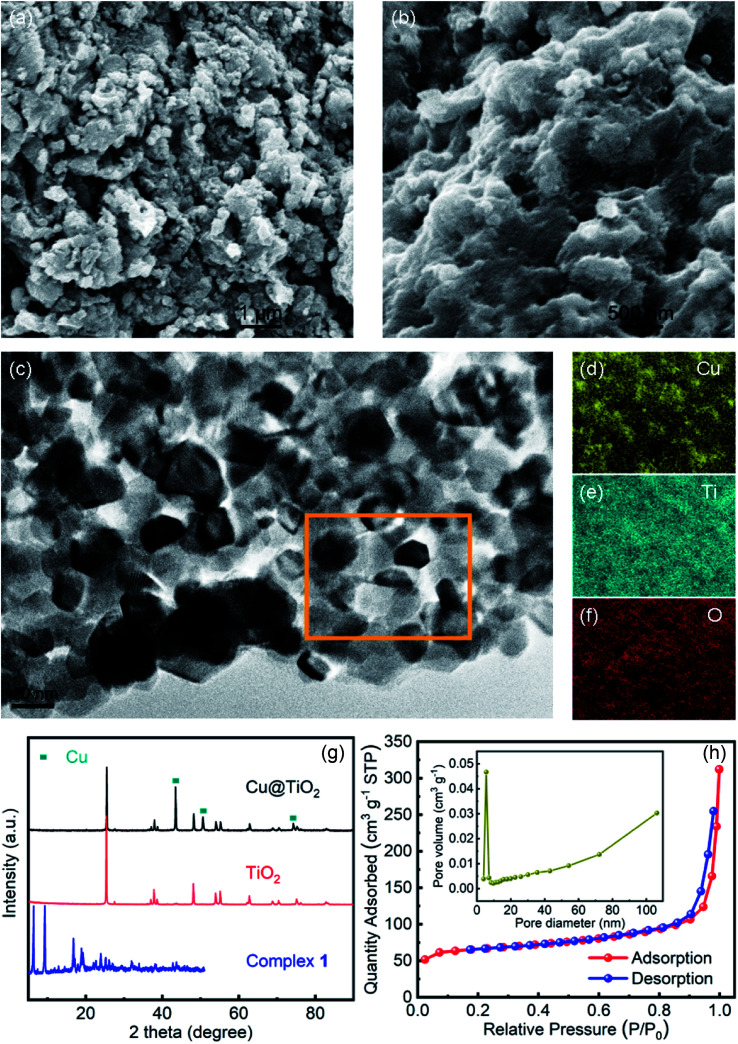
(a and b) SEM image of Cu@TiO_2_; HRTEM image (c) and the corresponding elemental maps of Cu (d), Ti (e) and O (f) within the square area of Cu@TiO_2_; (g) PXRD patterns of TiO_2_ and Cu@TiO_2_; (h) nitrogen adsorption and desorption isotherms of Cu@TiO_2_ (inset: the pore size distributions of Cu@TiO_2_).

The morphology of the Cu@TiO_2_ was observed by SEM and HRTEM characterization. After calcined, the Cu-CP was formed Cu nanoparticles, then the Cu nanoparticles were doped into TiO_2_. It can be seen that Cu@TiO_2_ maintains a flower-like lamellar microspheres with a diameter of 50–100 nm, and a large number of nanoparticles are loading on the surface of lamellar microstructures (Fig. 2a–c). Moreover, the nanoparticles in the lamellar matrix are well dispersed without exhibiting significant agglomeration. EDS mapping shows the element distribution of Cu, Ti and O in Cu@TiO_2_ (Fig. 2d–f). These characterizations demonstrate that most of TiO_2_ attached to the Cu nanoparticles surface are separately and evenly distributed. The PXRD pattern of the Cu@TiO_2_ material prepared *via* the calcine of Cu-CP is demonstrated in Fig. 2g. Cu@TiO_2_ exhibits three distinct maxima at 43.9°, 50.4°, and 74.2°, which are attributed to the (111), (200) and (220) planes of cubic Cu, respectively.^36^ Meanwhile, the Cu@TiO_2_ sample has a specific surface area of 219.85 m^2^ g^−1^ and a pore size distribution about 5.5 nm, suggesting that it possesses mesoporous structures (Fig. 2h). It is well known that the pores with variety of sizes is an appropriate way to increase active centers and can yield high-efficiency catalysts.^37^

**Fig. 3:**
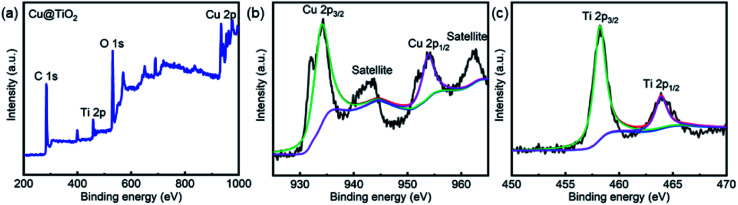
XPS spectra of Cu@TiO_2_: (a) XPS survey spectrum, (b) Cu 2p spectrum and (c) Ti 2p spectrum.

XPS studies were conducted over the Cu@TiO_2_ in order to understand the chemical state and chemical environment of the Cu and Ti elements of the composite. Fig. 3a shows the XPS survey spectrum for Cu@TiO_2_ composites, which demonstrates the existence of Cu, Ti and O elements in the sample. The spectra were calibrated with C 1s as standard. Fig. 3b shows the high resolution XPS spectrum in the region of Cu 2p for Cu@TiO_2_. The main peak centered at 934.3 and 953.8 eV of Cu (2p_3/2_) and Cu (2p_1/2_) are readily assigned to Cu(0).^38^ The two binding energy peaks with two extra shake-up satellites were assigned to Cu (2p_3/2_) and Cu (2p_1/2_) of Cu(ii).^38^ This was commonly attributed to the oxidation of Cu(0) during sample preparation for analysis as there is no CuO shown in the PXRD spectrum.^38^ The high resolution XPS spectrum of Ti 2p in Cu@TiO_2_ (Fig. 3c) centered in 458.3 and 464.0 eV can be assigned to Ti (2p_3/2_) and Ti (2p_1/2_) for Ti(iv).^39^

The UV-Vis diffuse reflectance spectroscopy was employed to investigate the optical properties of complex 1 and Cu@TiO_2_ samples. The UV-Vis absorption spectra of TiO_2_, complex 1, and Cu@TiO_2_ composite are shown in Fig. S4a.† It can be clearly seen that the pure TiO_2_ presents strong absorption of UV light at wavelengths below 400 nm. As desired, Cu@TiO_2_ composite exhibits much stronger absorption, especially in wavelengths ranging from 400 to 500 nm, which could be attributed to the interplay of Cu and TiO_2_ and the narrow band-gap of Cu. Using the Tauc plot of (*Ahn*)^2^*versus* photon energy (*hn*), the optical bandgap of the direct allowed semiconductor could be roughly determined.^40^ As shown in Fig. S4b,† the measured bandgap of pure TiO_2_ and the complex 1 is about 3.25 eV and 2.89 eV, respectively. As the coupling of Cu has a significant effect on the band-gap energy of TiO_2_, the estimated band-gap of the Cu@TiO_2_ composite is 2.54 eV, which is much lower than band-gap of either pure TiO_2_ or complex 1. These results indicate that the interfaces of Cu and TiO_2_ are combined intimately, and the band edges achieve good matching between the two semiconductors.

### 3.3 Dye photodegradation properties of Cu@TiO_2_ material

**Fig. 4:**
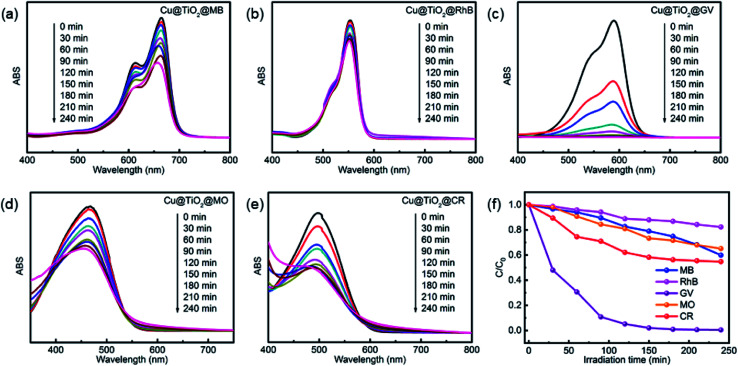
UV-Vis spectra of MB (a), RhB (b), GV (c), MO (d), and CR (e) solutions which were recorded after photocatalytic degradation had been performed for different lengths of time with Cu@TiO_2_. (f) The photodegradation rates of MB, RhB, MO, CR and GV at different time points during exposure to Cu@TiO_2_.

Herein, we evaluated the ability of complex 1 and Cu@TiO_2_ material to catalyze the photodegradation of five dyes under UV irradiation. Firstly, no significant change in the degradation of the five dyes was observed without catalysts (Fig. S5†). Moreover, it can be seen that the photocatalytic degradation rates are 11.15% for MB, 21.74% for RhB, 90.37% for GV, 69.05% for MO, and 58.70% for CR as well as 40.05% for MB, 17.66% for RhB, 99.51% for GV, 34.86% for MO, and 45.22% for CR after 4 h of UV irradiation for complex 1 and Cu@TiO_2_, respectively (Fig. 4 and S6†). Under UV light irradiation, an electron of O in the Cu@TiO_2_ could be induced transition from the highest occupied orbital (HOMO) to the lowest unoccupied orbital (LUMO) of the center ion. In order to be stable, the HOMO orbital had to capture an electron from a water molecule to produce a hydroxyl radical (˙OH), which has a strong oxidation ability and can selective degrade GV with rich N atoms.^40^ Moreover, the GV was completely degraded and converted into harmless products (CO_2_, H_2_O and inorganic molecules).^40–43^ Cui and co-workers have prepared PANI nanofibers loaded CP composite photocatalyst (PANI/CP1) through *in situ* chemical polymerization of aniline on the surface of Cd-CP.^44^ The photocatalytic degradation rate is 92.06% for RhB. Lang's group has report a ‘Ag(i)-doping’ synthetic strategy which have a better catalytic performance in the photodecomposition of azo dyes in industrial wastewater.^45^ These results demonstrated that Cu@TiO_2_ is highly efficient and selective toward GV and efficiently captures this dye. Moreover, to the best of our knowledge, there is no example in which the CP-derived metal-doped TiO_2_ materials did enhance the photodegradation performances of their parent materials under UV light (Table 2).

**Table 2:** Dye photodegradation activity of recently developed presentative CP-derived materials under UV light

Material	Dye	Degradation rate (%)	References
PANI/CP1	RhB	92.06	44
CP1 = [Cd(chdc)(4,4′-bipy)]_*n*_			
chdc = 4-cyclohexene-1,2-dicarboxylate			
4,4′-bipy = 4,4′-bipyridine			
{[Pb(Tab)_2_(bpe)]_2_(PF_6_)_4_·1.64AgNO_3_}_*n*_	MO	∼95	45
TabH = 4-(trimethylammonio)benzenethiol	CR	100	
bpe = 1,2-bis(4-pyridyl)ethylene			
Cu-CP-derived Cu@TiO_2_	MB	40.05	This work
	RhB	17.66	
	GV	99.51	
	MO	34.86	
	CR	45.22	

In kinetic experiments, the identification of the rate-determining step is of great importance in order to accurately predict which pathway is followed by the substrate as it binds with the catalyst. The experimental data obtained by photocatalysis experiments were fitted according to the pseudo-first-order kinetic model^46^ to calculate the values of rate constants and half-life periods and their correlation with the progress of the reaction. These calculations were performed *via*eqn (2) and (3):

2 ln(*C*_0_/*C*_*t*_) = *kt*

**Fig. 5:**
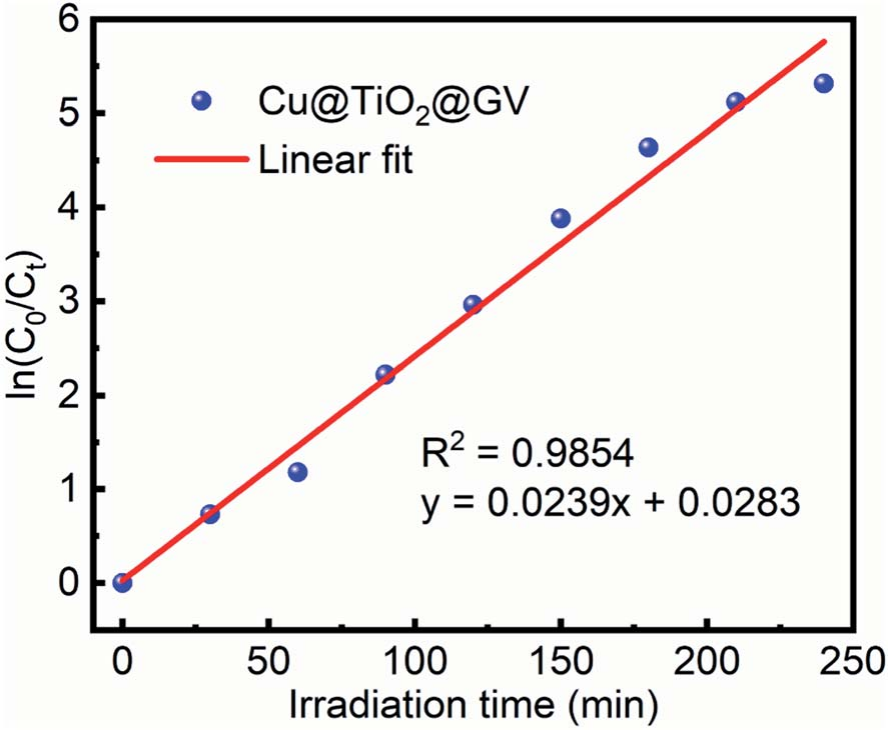
Pseudo-first-order plot with respect to time for Cu@TiO_2_ in an aqueous GV solution.

3
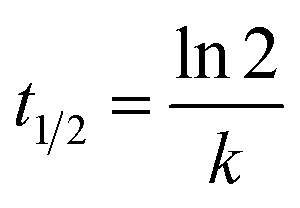
where *C*_0_ represents the initial concentration of GV (mg L^−1^), *C*_*t*_ is the residual concentration of GV at time *t* (min), *k* denotes the pseudo-first-order rate constant (min^−1^), and *t*_1/2_ (min) represents the half-life period of the reaction. Fig. 5 and S7† show that the degradation of the GV dye in the presence of Cu@TiO_2_ obeys pseudo-first-order kinetics. The slope of the straight line provided a pseudo-first-order rate of 0.0239 min^−1^ and a *t*_1/2_ value of 29.00 min for the degradation of GV by Cu@TiO_2_ (Table 3).

**Table 3:** Pseudo-first-order kinetic parameters for the photodegradation of GV by Cu@TiO_2_

Concentration (mg L^−1^)	Rate constant (K min^−1^)	Half-life *t*_1/2_ (min)	*R* ^2^
10	0.0239	29.00	0.9854

**Fig. 6:**
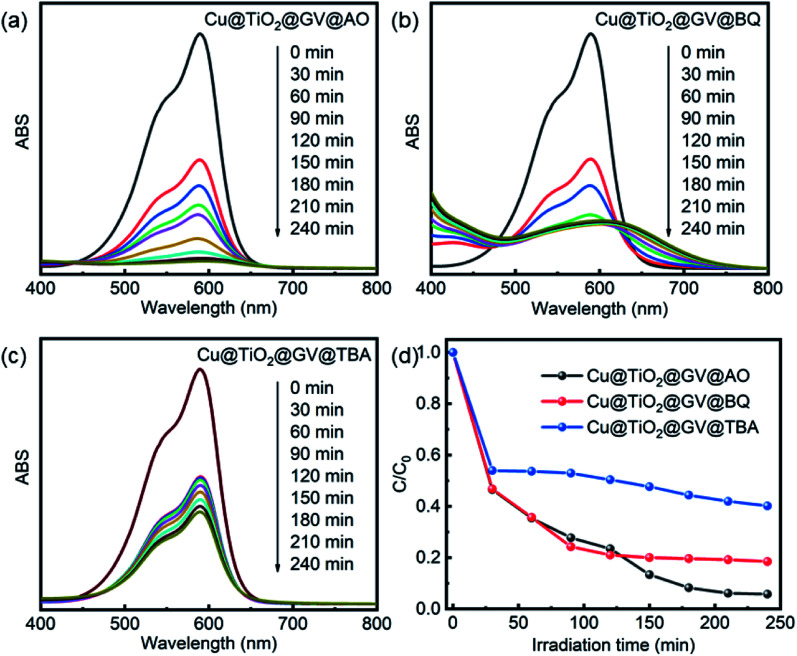
UV-Vis spectra of GV solution on the Cu@TiO_2_ in presence of AO (a), TBA (b) and BQ (c). (d) The photodegradation rates of GV on the Cu@TiO_2_ in presence of various scavengers.

Through comparison of the degradation of the dyes by Cu@TiO_2_ under similar exposure except for the presence or absence of a UV light (Fig. 6), it had been inferred that h^+^, ˙OH, or ˙O_2_^−^ play a key role in the degradation of aromatic dyes. The photocatalytic reaction mechanism can be explained by eqn (4)–(8):

4 Cu@TiO_2_ + *hv* → h^+^ + e^−^

5 h^+^ + OH^−^ → ˙OH

6 h^+^ + H_2_O → ˙OH + H^+^

7 e^−^ + O_2_ → ˙O_2_^−^

8 ˙O_2_^−^ or ˙OH + dye → degraded + products

When the photon energy is greater than the width of Cu@TiO_2_ forbidden band, electronic transitions will proceed from the valence band to the conduction band, thus forming the electrons and cavities. On the one hand, the photogenerated holes (h^+^) were ultimately trapped by surface hydroxyl groups and H_2_O on the catalyst surface to promote the oxidation and enhance the reactivity of the hydroxyl radicals (˙OH) as shown in eqn (5) and (6). On the other hand, the photogenerated electrons react with the O_2_ adsorbed on the surfaces of Cu@TiO_2_ to form oxygen radicals (˙O_2_^−^), as shown in eqn (7). Notably, ˙O_2_^−^ and ˙OH are two main active species involved in the overall process. As powerful oxidants, ˙O_2_^−^ and ˙OH can promote the decomposition of the organic dye as shown in eqn (8).

In the photocatalytic oxidation (PCO) process, a series of reactive oxygen species, such as h^+^, ˙OH, or ˙O_2_^−^, are supposed to be involved. Ammonium oxalate (AO), *tert*-butyl alcohol (TBA) and benzoquinone (BQ) were scavengers of h^+^, ˙OH, or ˙O_2_^−^, respectively.^40^ In order to investigate high-efficiency of the as-prepared Cu@TiO_2_ for the degradation of GV, we conducted the reactive species trapping experiments at optimized conditions after irradiation for 240 min and the corresponding results are shown in Fig. 6. Without using any scavenger, the degradation of GV on the Cu@TiO_2_ was found to be 99.51%. In the presence of TBA and BQ, the degree of degradation diminished to 59.79% and 76.09%, respectively. It can be seen that the addition of AO in the GV solution has little effect on the photocatalytic activity of Cu@TiO_2_, suggesting that h^+^ does not play a key role for the degradation of GV.^47–49^ On the contrary, the photocatalytic degradation of GV is obviously inhibited after the addition of TBA and BQ. On the basis of these results, it can be concluded that ˙OH and ˙O_2_^−^ are the main oxygen active species for Cu@TiO_2_ in the GV solution under UV light irradiation.

Moreover, control experiments were carried out for the title Cu@TiO_2_. The pure TiO_2_ was added to the GV solution under UV irradiation (Fig. S8†). However, the photodegradation rate of dyes in presence of Cu@TiO_2_ was higher than in presence of pure TiO_2_ and Cu-CP (Fig. S6†), and the good dispersion of Cu on TiO_2_ accelerated the separation of ˙OH, or ˙O_2_^−^, which made Cu@TiO_2_ have high photocatalytic activity.^50^ Thus, these features demonstrate that the title Cu@TiO_2_ can be a highly effective sorbent for the selective removal of dye species from water and can catalyze their decomposition upon exposure to UV light.

The cyclic photodegradation performance of the Cu@TiO_2_ was studied with a constant GV concentration (10 mg L^−1^) and amount of adsorbent (5 mg). After each cycle (240 min) of GV photodegradation, the recovered Cu@TiO_2_ was dipped into ethanol and dried at 50 °C and reused for photodegradation in the following cycles. There is no obvious adsorption decrease observed after 10 cycles (Fig. S9†), indicating a stable photodegradation ability of the Cu@TiO_2_. Furthermore, PXRD observation shows that the structure of the Cu@TiO_2_ was well preserved after each cycle (Fig. S10†).

## 4. Conclusions

*In situ* Cu@TiO_2_ composites were successfully synthesized by annealing Cu-CP at low temperature. Cu@TiO_2_ shows twice higher photocatalytic GV degradation rate than the TiO_2_ under UV light irradiation. This excellent photocatalytic performance is closely related to the morphology, crystallinity and structure of the catalysts. The unique porous structure from CP can enhance the light collection through the reflection effect. In addition, the co-doping of Cu can accelerate the separation of photogenerated charges and reduce the band gap. Furthermore, ˙OH and ˙O_2_^−^ were the main reactive species, which are responsible for the GV degradation. This work presents a facile and versatile strategy for the preparation of photocatalysts that hold great promise in the treatment of organic wastes for environmental remediation.

## Conflicts of interest

The authors declare no competing financial interest.

## Supplementary Material

RA-012-D1RA09309F-s001

RA-012-D1RA09309F-s002
