# Supplementary material for: Synthesis of a 2D Cu@TiO2 composite via the design of a 1D Cu-based coordination polymer precursor for efficient and selective photodegradation of dyes
Source: RSC Adv. 2022 Mar 24;12(15):9363–72. doi: 10.1039/d1ra09309f (PMC8985212; doi:10.1039/d1ra09309f)
Supplement: RA-012-D1RA09309F-s001 [file RA-012-D1RA09309F-s001.pdf]

Electronic Supplementary Material (ESI) for RSC Advances.
This journal is © The Royal Society of Chemistry 2022

Supplementary Material (ESI)

Synthesis of a 2D Cu@TiO_2 composite via the design of a 1D Cu-based coordination polymer precursor for efficient and selective photodegradation of dyes

Ya-Qian Zhang,[a, c] Ning Xu,[a] Yu Liu,[a] Xiao-Sa Zhang,[a] Wen-Ze Li[a*], Hong-Tian Zhao[a] and Jian Luan[b*]

[a] *College of Science, Shenyang University of Chemical Technology, Shenyang, 110142, P. R. China*

[b] *College of Sciences, Northeastern University, Shenyang, 100819, P. R. China*

[c] *College of Chemistry, Liaoning University, Shenyang 110036, P. R. China*

E-mail: liwenze@syuct.edu.cn (W. Z. Li); 2010044@stu.neu.edu.cn (J. Luan)

Table S1 Selected bond distances (Å) and angles (°) for complex **1**.

Cu(1)–O(1)	1.9265(17)	Cu(1)–O(4)#1	1.9441(16)
Cu(1)–O(1W)	1.9489(16)	Cu(1)–N(1)	1.988(2)
Cu(2)–O(8)#2	1.9581(17)	Cu(2)–O(3W)	1.9818(17)
Cu(2)–O(5)	1.9868(16)	Cu(2)–N(2)	2.029(2)
Cu(2)–O(2W)	2.2214(18)	O(1)–Cu(1)–O(4)#1	165.73(8)
O(1)–Cu(1)–O(1W)	89.36(7)	O(4)#1–Cu(1)–O(1W)	87.93(7)
O(1)–Cu(1)–N(1)	91.43(7)	O(4)#1–Cu(1)–N(1)	92.36(8)
O(1W)–Cu(1)–N(1)	175.54(8)	O(8)#2–Cu(2)–O(3W)	89.79(7)
O(8)#2–Cu(2)–O(5)	179.73(8)	O(3W)–Cu(2)–O(5)	90.47(7)
O(8)#2–Cu(2)–N(2)	89.28(7)	O(3W)–Cu(2)–N(2)	171.84(8)
O(5)–Cu(2)–N(2)	90.47(7)	O(8)#2–Cu(2)–O(2W)	90.46(7)
O(3W)–Cu(2)–O(2W)	89.66(7)	O(5)–Cu(2)–O(2W)	89.49(7)
N(2)–Cu(2)–O(2W)	98.45(8)		

Symmetry codes: #1 $x, y - 1, z$; #2 $x, y + 1, z$.

Table S2 Hydrogen bonding geometries (Å, °) of complex **1**.

D–H···A	D–H	H···A	D···A	D–H···A
N3–H3B···O5[a]	0.86	2.17	3.0036	162
O3W–H3WB···O3[b]	0.85	1.94	2.7628	162
O2W–H2WA···O9[c]	0.85	2.14	2.7205	125

Symmetry codes: [a] $1 - x, -y, 1 - z$; [b] $x, -1 + y, -1 + z$; [c] $1/2 + x, 1/2 - y, -1/2 + z$.

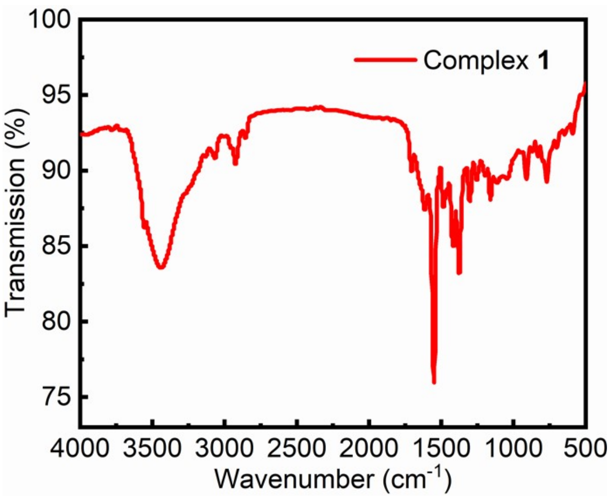

Fig. S1 The IR spectrum of complex **1**.

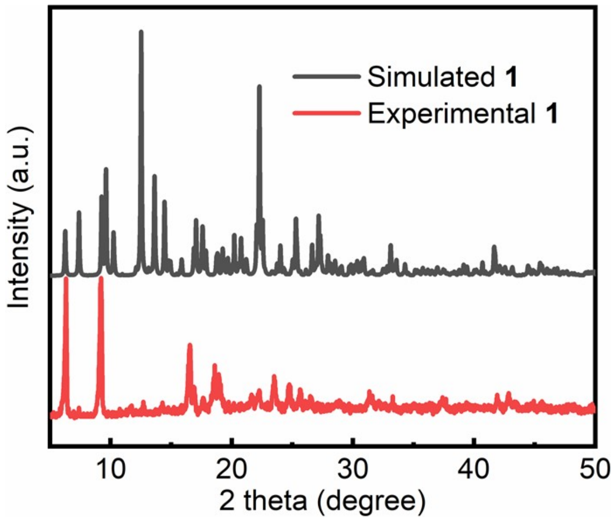

Fig. S2 The PXRD patterns of simulated and fresh sample for complex **1**.

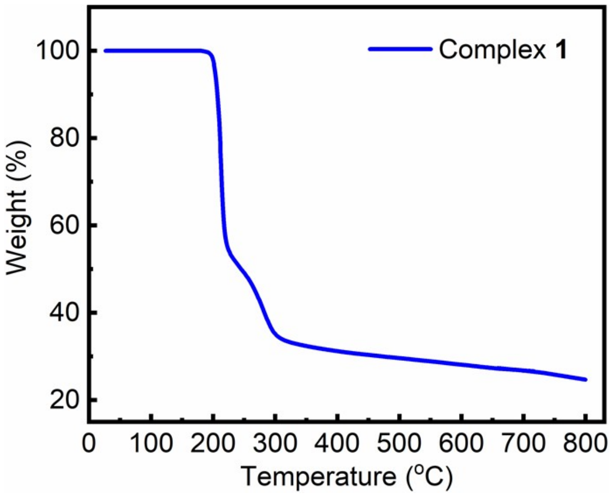

Fig. S3 The TG curve of complex **1**.

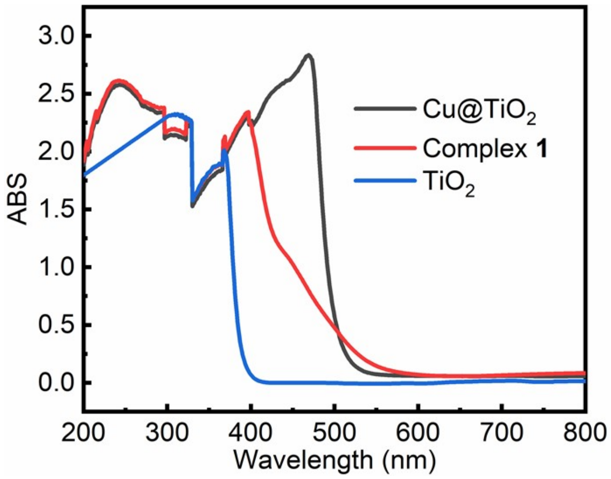

(a)

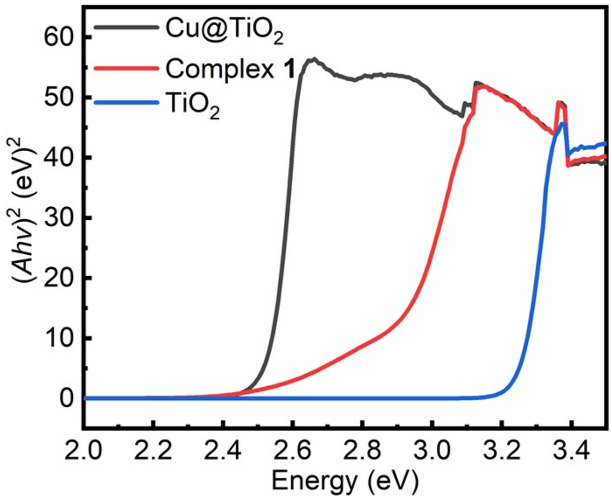

(b)

Fig. S4 UV-vis diffuse-reflectance spectra (a) and Tauc plots of $(Ah\nu)^2$ versus $(h\nu)$ (b) of TiO_2, complex **1**, and Cu@TiO_2, respectively.

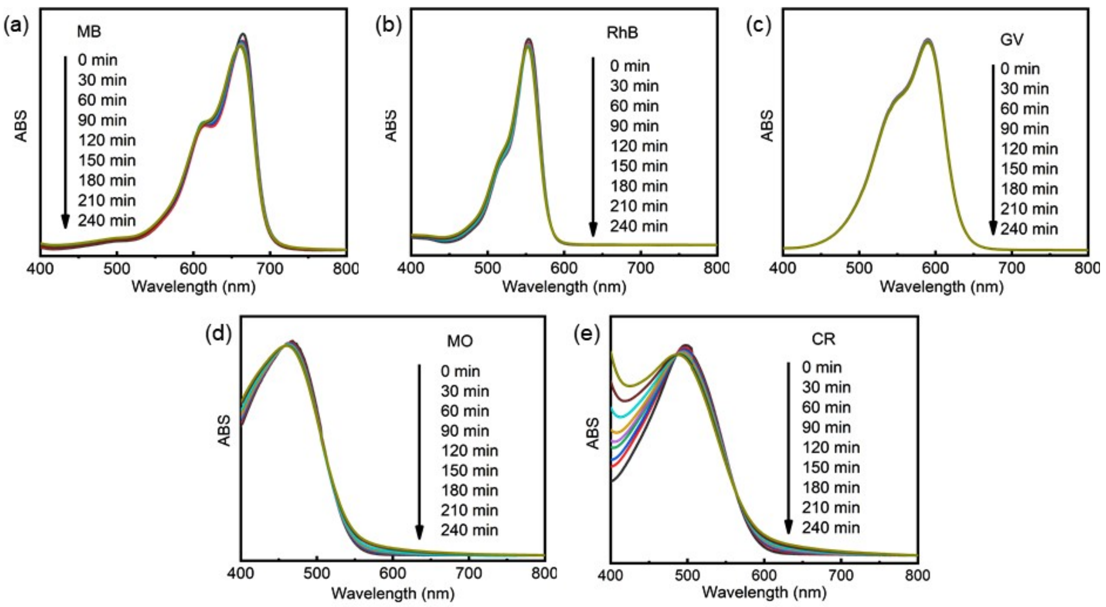

Fig. S5 UV–vis spectra of MB (a), RhB (b), GV (c), MO (d), and CR (e) solutions which were recorded after photocatalytic degradation had been performed for different lengths of time without catalysts.

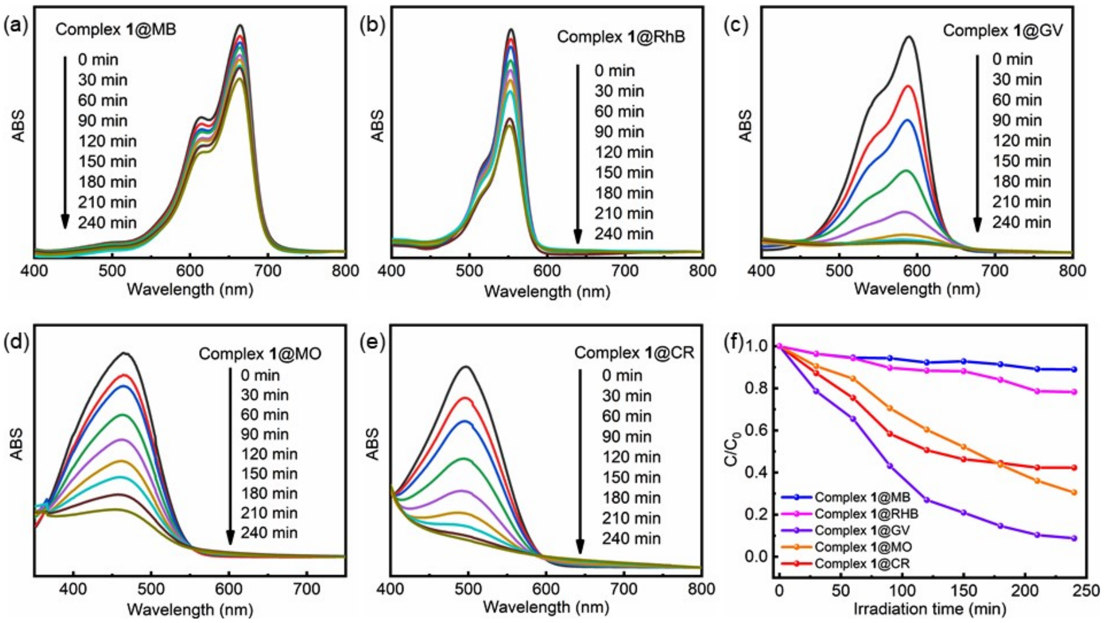

Fig. S6 UV–vis spectra of MB (a), RhB (b), GV (c), MO (d), and CR (e) solutions which were recorded after photocatalytic degradation had been performed for different lengths of time with complex **1**. (f) The photodegradation rates of MB, RhB, MO, CR and GV at different time points during exposure to complex **1**.

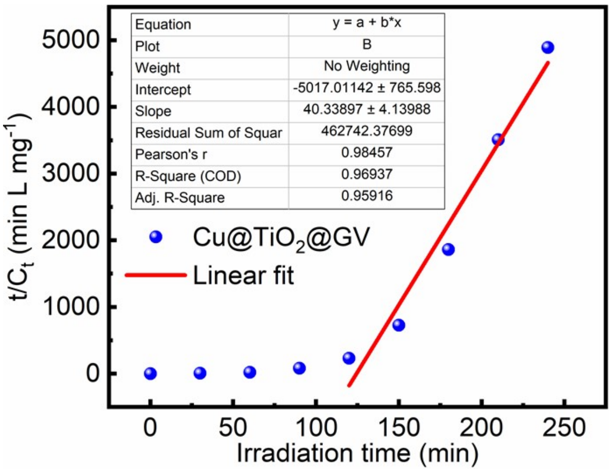

Fig. S7 Pseudo-second-order plot with respect to time for Cu@TiO_2 in an aqueous GV solution for 90–240 min under UV irradiation. Equation: $t/C_t = 1/(kC_t^2) + t/C_0$, where C_0 represents the initial concentration of GV (10 mg L^{-1}), C_t is the residual concentration of GV at time t (min), and k denotes the pseudo-second-order rate constant (L mg^{-1} min^{-1}).

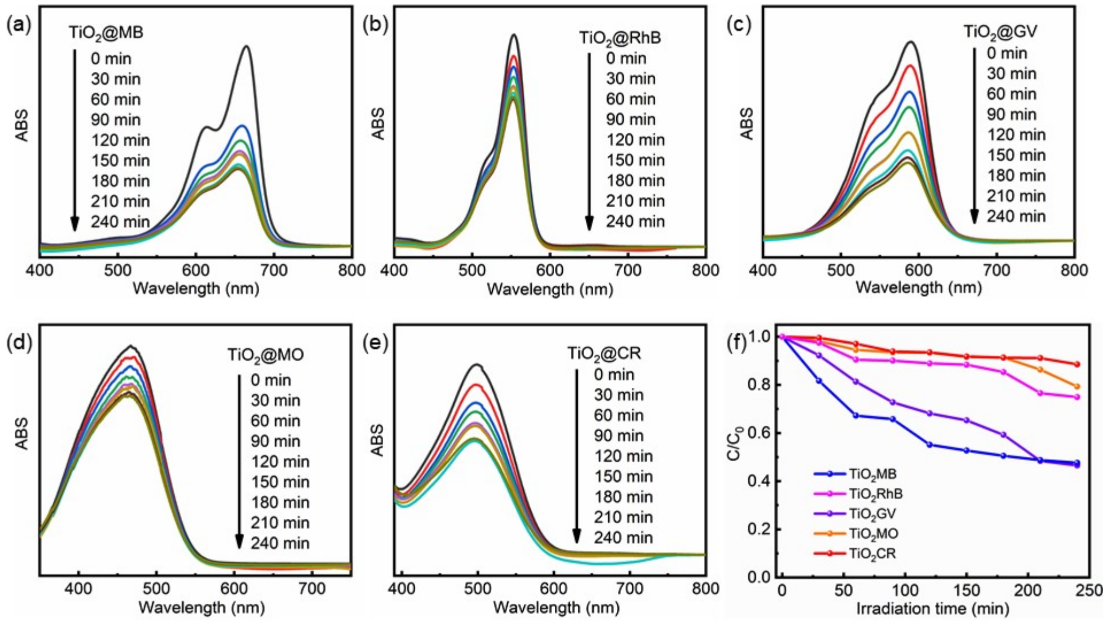

Fig. S8 UV–vis spectra of MB (a), RhB (b), GV (c), MO (d), and CR (e) solutions which were recorded after photocatalytic degradation had been performed for different lengths of time with pure TiO_2. (f) The photodegradation rates of MB, RhB, MO, CR and GV at different time points during exposure to pure TiO_2.

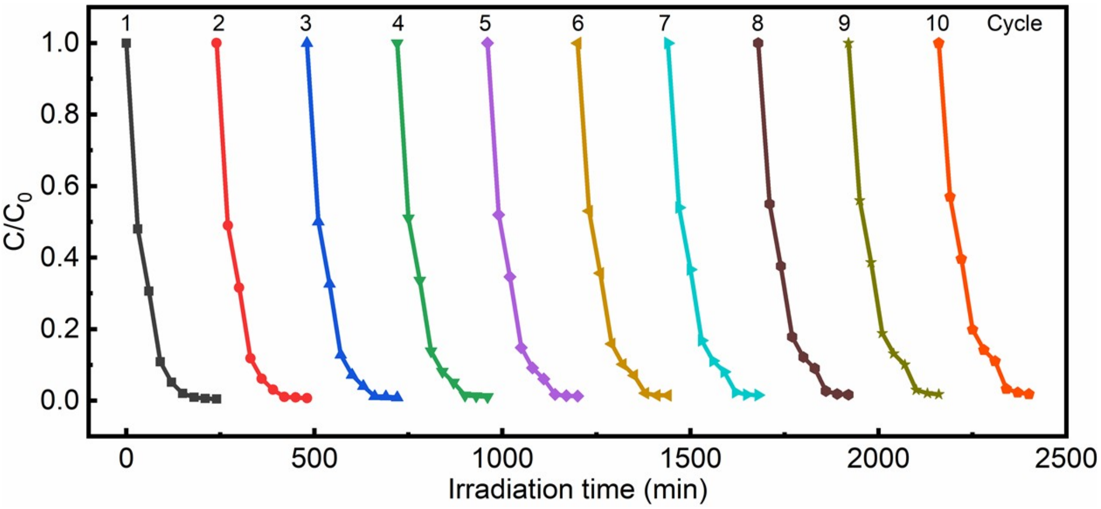

Fig. S9 The cycling stability of the photocatalytic degradation of GV on the Cu@TiO_2.

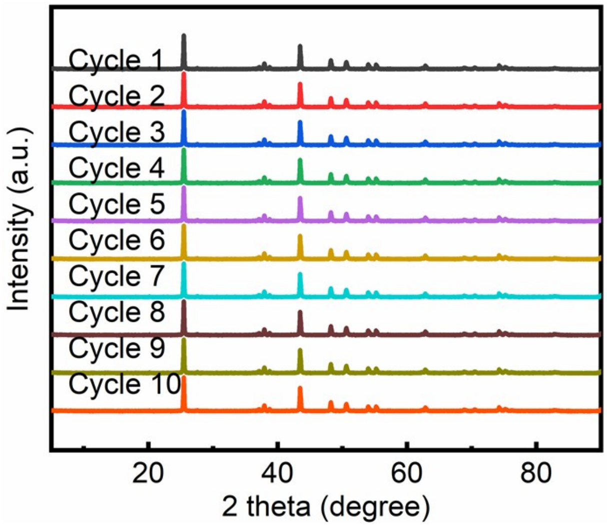

Fig. S10 The PXRD patterns of the cycling stability of the photocatalytic degradation of GV on the Cu@TiO_2.
